# Long-Term Fracture Resistance of Simulated Immature Teeth Filled with Various Calcium Silicate-Based Materials

**DOI:** 10.1155/2016/2863817

**Published:** 2016-06-13

**Authors:** Yeliz Guven, Elif Bahar Tuna, M. Emir Dincol, Emre Ozel, Bulent Yilmaz, Oya Aktoren

**Affiliations:** ^1^Department of Pedodontics, Faculty of Dentistry, Istanbul University, 34093 Istanbul, Turkey; ^2^Department of Endodontics, Faculty of Dentistry, Istanbul University, 34093 Istanbul, Turkey; ^3^Department of Restorative Dentistry, Faculty of Dentistry, University of Kocaeli, 41190 Kocaeli, Turkey

## Abstract

*Objective*. The aim of this in vitro study was to evaluate the long-term fracture resistance of simulated human immature permanent teeth filled with BioAggregate*™* (BA), mineral trioxide aggregate (MTA), and EndoSequence® Root Repair Material (ERRM).* Material and Methods*. 40 teeth, simulated to average root length of 13 ± 1 mm (Cvek's stage 3), were included in the study. The teeth were randomly divided into four groups: Group 1:* DiaRoot*® BA, Group 2: MTA-Plus*™* (MTA-P), Group 3: MTA-Angelus (MTA-A), and Group 4: ERRM. The root canal filling materials were applied according to the manufacturers' instructions. After 24 months of incubation, the roots of the teeth were embedded in acrylic blocks and subjected to fracture testing. The resultant data were analyzed statistically by Kruskal-Wallis and Mann-Whitney *U* tests.* Results*. Mean (±SD) failure loads (MPa) were 20.46 ± 2.53 for BA, 18.88 ± 5.13 for MTA-P, 14.12 ± 1.99 for MTA-A, and 17.65 ± 4.28 for ERRM groups. BA group exhibited the highest and MTA-A group showed the lowest resistance to fracture. Significant differences in fracture resistance were found between the groups of BA and MTA-A (*p* < 0.001), MTA-P and MTA-A (*p* < 0.05), and ERRM and MTA-A (*p* < 0.05).* Conclusion*. Within the limitations of this study, data suggests that BA-filled immature teeth demonstrate higher fracture resistance than other groups at 24 months appearing to be the most promising material tested.

## 1. Introduction

Traumatic dental injuries are frequent in children aged 8–12 years, and the maxillary incisors are the most commonly affected teeth. A traumatic impact on the immature anterior teeth frequently results in arrested root development due to the loss of pulp vitality. The endodontic treatment of these teeth with necrotic pulps poses a challenge for the practitioner because of the widely open apices and the thin dentinal walls which predispose teeth to fracturing [1, 2]. Root fractures commonly occur in the cervical third and have been shown to have a rate of about 28–77%, depending on the stage of root development, with the highest percentage of fractures occurring in teeth with the least developed roots [3].

An optimal approach to treating the immature permanent tooth with a necrotic pulp would be to regenerate functional pulpal tissue and subsequently promote continued root development and apical closure [4]. A regenerative endodontic procedure, revascularization, was presented recently to treat immature permanent teeth. Although it has demonstrated great potential for clinical success, it needs to be evaluated over the long term, and it may not be successful in every case [5]. Accordingly, apexification through the use of calcium hydroxide (CH) or mineral trioxide aggregate (MTA) still represents the most widely indicated treatment for necrotic cases of immature teeth. CH apexification by means of the induction of an apical barrier and the antibacterial capacity of the agent—due to its high pH—has been used for more than 50 years and has proved to be successful in apical healing [6]. However, long-term application of CH has been reported to possess numerous disadvantages, including long duration of treatment and requirement for multiple appointments. The risk of fractures between appointments and the possibility of recontamination due to the dislodgement of temporary filling are additional shortcomings of CH apexification. Single visit apexification using MTA has recently been advocated to overcome these difficulties inherent in long-term CH treatment, and it has resulted in favorable clinical outcomes [7, 8]. Immediate apical barrier formation using MTA offers several advantages over conventional apexification, such as reduced risk of subsequent cervical root fracture and increased patient compliance. Aside from these advantages, however, MTA is difficult to handle and has a long setting time, and its high cost restricts its clinical application [7, 9].

The search for bioceramic materials exhibiting properties similar to MTA but with improved handling characteristics and shorter setting times has led to the development of newer products which have been referred to as calcium silicate-based cements. BioAggregate (BA) (Innovative BioCeramix Inc., Vancouver, BC, Canada), a tricalcium silicate-based and aluminum-free ceramic biomaterial, has been developed for use in retrograde root filling, repair of root perforation, apexification, vital pulp therapy, and pulp capping [9, 10]. EndoSequence Root Repair Material (ERRM) has recently been introduced to the dental market. The material uses bioceramic technology and is produced in a premixed state, either with a ready-to-use syringeable paste or compactable putty, both of which provide easier handling and application than MTA. ERRM is composed of zirconium oxide, calcium silicates, tantalum oxide, calcium phosphate monobasic, and filler agents [11]. Although all calcium silicate-based materials induce clinically perceptible color changes, BA and ERRM are also advantageous since they exhibit less discoloration than MTA [12, 13].

Calcium silicate-based materials have been proposed as a promising alternative to CH in apexification procedures because of their high biocompatibility and their superior ability to set in the presence of moisture. Despite favorable clinical and laboratory outcomes obtained when using these calcium silicate-based materials to treat teeth with immature apexes, the high rate of cervical root fractures in the long term is still a significant concern. Accordingly, ongoing efforts have been directed towards searching for materials with improved biological and mechanical properties. Furthermore, there has been limited information on the strengthening capacity of novel root canal filling materials. In this regard, the aim of this in vitro study was to assess the long-term fracture strength of simulated human immature permanent teeth filled with BA, MTA, and ERRM.

## 2. Materials and Methods

### 2.1. Tooth Selection

Extracted human maxillary central incisor teeth, thoroughly hand-scaled and cleaned with water/pumice slurry using a low-speed handpiece, were stored in distilled water at 4°C until use. All teeth were examined under ×4 magnification, and only intact teeth without cracks, fractures, or caries were included in the study. Preoperative radiographs were taken in the faciolingual and mesiodistal directions to confirm the presence of a single canal without resorptions or calcifications. The selected teeth were measured with a digital caliper in the buccolingual and mesiodistal dimensions at the cementoenamel junction (CEJ) and the mean values were obtained. Teeth displaying more than 10% deviation were excluded, leaving 40 teeth for use in the study. All procedures were performed by the same experienced operator.

The root of each tooth was standardized to a length of 13 ± 1 mm as measured from the apex to the facial CEJ by cutting off the root end to simulate immature teeth (Cvek's stage 3). The length of the specimens was measured by digital caliper.

### 2.2. Treatment Procedures

Coronal access was made using a #10 round diamond bur (Strauss & Co., Industrial Diamonds Ltd. Ra'anana, Israel) and endo-Z bur (Dentsply Maillefer, Ballaigues, Switzerland) in a high speed handpiece. The pulps were extirpated using barbed broaches (DiaDent Group Int., Canada). To simulate immature teeth, the root canals were instrumented with Peeso reamers (Dentsply Maillefer, Ballaigues, Switzerland) between #1 and #6 until a size 6 Peeso (1.7 mm) could be passed 1 mm beyond the apex. The canals were irrigated with 5 mL 5.25% sodium hypochlorite (NaOCl) (Sultan Chemists Inc., Englewood, USA), 5 mL 17% EDTA (Pulpdent Corporation, Watertown, MA, USA), and 5 mL. 0.9% saline, respectively. During all of the procedures, teeth were wrapped in moistened gauze. After instrumentation and irrigation, the canals of the teeth were filled with the root canal filling materials according to manufacturers' instructions.

**Figure 1:**
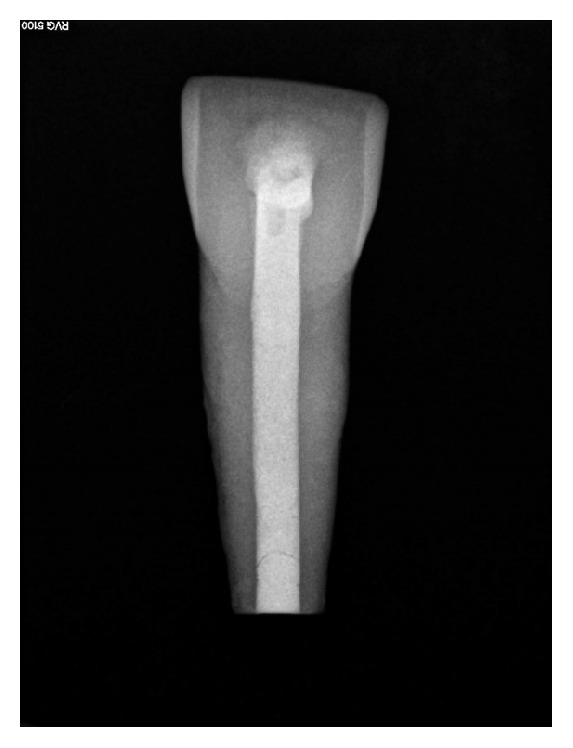
Frontal view of the simulated immature teeth after root filling.

Manufacturers and composition of the materials used in the study are summarized in Table 1.  Figure 1 shows the radiographic appearance of a representative sample of an immature tooth obturated with respective root canal filling material.

The teeth were randomly assigned to four groups of ten teeth each, given as follows.

*Group 1* (DiaRoot BA (BA) (DiaDent Group International, Canada)). 1 g of BA powder was mixed with 0.38 mL of liquid included in the package according to the manufacturer's instructions. Teeth were filled using lentulo spiral (Mani Inc., Tochigi-Ken, Japan) and pluggers. The apices and the coronal parts were covered with a moist cotton pellet for 12 hours before placing the permanent restoration.

*Group 2* (MTA-Plus (MTA-P) (Avalon Biomed Inc., Prevest Denpro Limited, India)). The root canal was filled with MTA-P which is provided with either water or a gel for mixing. In the present study, it was mixed with water. The paste was carried to the coronal part of the pulp cavity using a lentulo spiral, and it was condensed using pluggers. The manufacturer indicates no specific requirement for moisture to allow finalization of the setting reaction.

*Group 3* (MTA-Angelus (MTA-A) (Angelus Soluções Odontológicas, Londrina, PR, Brazil)). The white MTA-A was placed in the root canal using a lentulo spiral and then condensed using pluggers. The moisture is not required for setting reaction.

*Group 4* (EndoSequence Root Repair Material (ERRM) (Brasseler, USA)). ERRM was introduced into the entire root canal using the manufacturer-provided preloaded syringe with the delivery tip. The manufacturer does not indicate any moisture-specific instructions to allow finalization of the setting reaction. 

All specimens were radiographed from lateral and facial views of the teeth after the root treatment to verify the filling density. In all groups, after the radiographic confirmation, the coronal access of each specimen was restored with a glass ionomer base (Fuji II LC; GC America, Inc.) and composite resin filling (Filtek Z250, 3M ESPE, St. Paul, MN, USA). The coronal access of all teeth was restored with glass ionomers (Fuji II LC; GC America Inc.), followed by composite resins (Filtek Z250, 3M ESPE, St. Paul, MN, USA). All specimens were stored in 100% humidity at 37°C for 24 months until fracture resistance testing.

### 2.3. Fracture Strength Test

**Figure 2:**
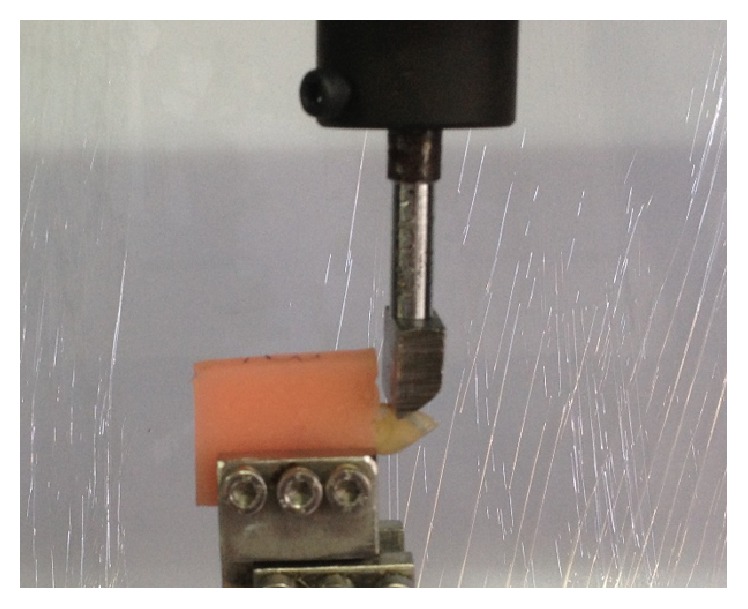
Compression force was applied at a point 3 mm from the CEJ and perpendicular to the long axis of the tooth with an Instron Universal Testing Machine.

The root of each tooth was embedded vertically in self-curing orthodontic resin blocks (Dentsply, Tulsa, OK) with dimensions of 27 mm × 15 mm × 13 mm. The long axis of each tooth was aligned with the central axis of the acrylic resin block. The roots were submerged in acrylic resin, leaving a 2 mm gap between the CEJ and the top of the resin to simulate the relation between the tooth and the bone crest. The spade was placed on the facial surface at a point 3 mm above the CEJ, and loading was applied perpendicular to the specimen's long axis at a cross head speed of 1 mm/min in a universal testing machine (Instron, AG-IS, Shimadzu, Japan) until the initial fracture occurred (Figure 2). The maximum load at which the samples fractured was recorded in Newtons (N), and the fracture strength (force/area) was calculated in MPa.

### 2.4. Statistical Analysis

The mean and standard deviation (SD) for each group was calculated. The findings were analyzed statistically using a Kruskal-Wallis test to detect any intergroup differences and by means of the Mann-Whitney *U* test to evaluate comparisons at a 5% level of significance.

## 3. Results

The mean fracture strength values and SD for all groups are shown in Table 2. Mean (±SD) failure loads (MPa) were 20.46 ± 2.53 for BA, 18.88 ± 5.13 for MTA-P, 14.12 ± 1.99 for MTA-A, and 17.65 ± 4.28 for ERRM groups. All specimens showed either oblique or horizontal fractures through the cervical area of the root.

**Figure 3:**
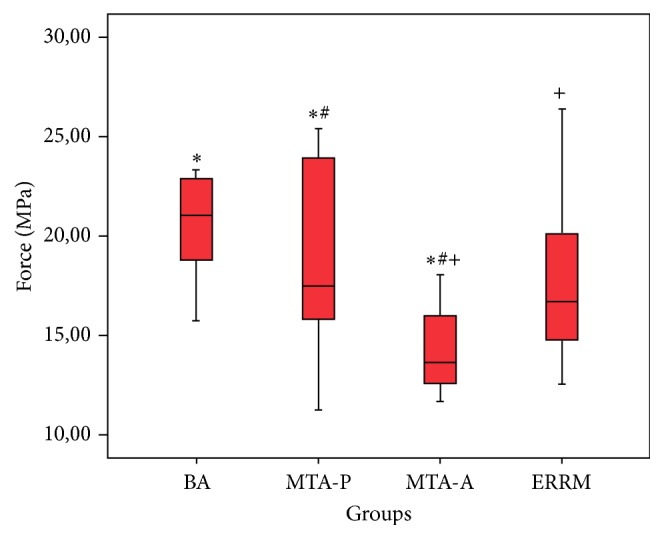
Boxplots with forces required to cause cervical root fracture for each of the groups. *∗*, #, and + symbols represent significant differences (*p* < 0.05).

Significant differences were found in failure loads among all tested groups according to the results of the Kruskal-Wallis analysis of variance (*p* < 0.01). The BA group exhibited the highest and the MTA-A group showed the lowest resistance to fracture. Significant differences in fracture resistance were found between the BA and MTA-A and MTA-Plus and MTA-Angelus and also between ERRM and MTA-A groups (*p* < 0.05) (Figure 3). The Mann-Whitney *U* test revealed no other significant differences (*p* > 0.05) between the other groups (Table 2).

## 4. Discussion

Endodontically treated immature teeth are susceptible to fracture depending on their stage of root development, which is directly related to the remaining dentin wall thickness and root length [3]. It is well established that as the dentin wall thickness decreases, the resistance to fracture decreases as well, and therefore it becomes more important to select a material that has potential to reinforce the root structure [5, 14]. A number of studies have focused on increasing fracture resistance of immature teeth using various techniques [15–19]. MTA has been a revolutionary material in endodontics because of its high biocompatibility and sealing ability. Due to the promising results obtained with MTA, a new generation of endodontic materials with similar composition to MTA, but with some modifications aimed at overcoming the current drawbacks of the original material, has been developed and named calcium silicate-based cements because of their primary components of calcium and silicate [20, 21]. This in vitro study was conducted to investigate the long-term fracture strength of simulated human immature permanent teeth filled with three different calcium silicate-based materials: MTA, BA, and ERRM.

The methods for the simulation of immature teeth and preparation of the models for the fracture strength tests differed widely among previous studies [22]. In those studies, the analysis of the fracture resistance has been tested using sheep [1, 23] or bovine teeth [18, 24] or simulated human teeth [5, 19, 25, 26] or immature teeth extracted for orthodontic purposes [16]. This study used a simulated immature tooth model similar to that of Hemalatha et al. [26].

The mature teeth used in the present study may simulate the morphology of immature teeth, but they may not simulate the tissue composition and physical characteristics exactly, and this may be considered a limitation of these kinds of studies as stated by Ulusoy et al. [22]. Nevertheless, all of the experimental teeth went through the same procedures for the simulation of immature teeth; therefore, it can be assumed that they still allow for a relative comparison for assessing the resistance of immature teeth to fracture [17]. Only maxillary central incisors were used in the present study, as they are more susceptible to trauma and external impacts owing to their localizations.

Previous studies have evaluated the fracture resistance of immature teeth restored by various root canal filling systems, such as composite resin, fiber post, gutta-percha [1, 27], MTA [16], and recently introduced calcium silicate-based materials including Biodentine [19] and BA [16].

The nanosphere structure of ERRM particles allows the material to penetrate into the dentinal tubules and interact with the moisture inside the tubules for final setting. This creates a mechanical bond with dentine upon setting and renders the material with exceptional dimensional stability [28, 29]. The material has additional properties such as a high alkaline pH, radiopacity, hydrophilic setting properties, and ideal working (more than 30 min) and setting time [28]. These properties of ERRM have led to consideration of its usage for repair of root perforations, root-end fillings, pulp capping, pulpotomy, and root canal obturation [20]. Currently, there is very limited research on ERRM regarding its usage on root canal obturation. It has mainly been evaluated for use as a root-end filling material. Furthermore, there is no data regarding the fracture resistance of teeth filled with ERRM. In the present study, ERRM has been investigated for the first time with regard to its fracture resistance and has been found to have a significantly higher fracture resistance than MTA-A after 2 years. No significant difference was observed when compared to BA and MTA-P.

BA is composed of fine nanoparticle-size, aluminum-free powder that is mixed with deionized water to form a bioceramic paste [20]. BA contains hydroxyapatite, which has been proposed as an addition to root-end filling materials in order to enhance their ability to form a biochemical bond to the bone [30].

Tuna et al. assessed the long-term fracture resistance of human immature permanent teeth filled with BA, MTA, and CH and reported that the immature teeth filled with BA showed the highest fracture resistance in all groups, although statistically significant difference was observed only between BA and CH groups [16]. The results of the present investigation correlate well with those reported by Tuna et al. [16]. In the current study, the BA group showed higher resistance to fracture than other groups, although this difference only reached statistical significance on comparison with MTA-A group. The high fracture resistance of BA may be attributed to the absence of calcium hydroxide in the aged cement considering the negative long-term effects of calcium hydroxide on the fracture susceptibility of the root. Camilleri et al. investigated the effects of additives on the hydration mechanism of BA by characterization of the unhydrated and hydrated forms, using a combination of techniques and comparing these properties to MTA-Angelus [31]. Tricalcium silicate in BA results in calcium silicate hydrate and calcium hydroxide following setting reactions. The former was deposited around the cement grains, while the latter reacted with the additive in BA, silicon dioxide, to form additional calcium silicate hydrate. This resulted in very low levels of calcium hydroxide in the aged cement thus enhancing the mechanical properties of the cement. MTA-A reacted in a similar fashion; however, since it contained no additives, the calcium hydroxide was still present in the aged cement [31].

Variations on the formulation have been made to overcome the drawbacks associated with MTA. MTA-A and MTA-P are two of those products. MTA-A exhibits a reduced setting time as a result of a lower concentration of calcium sulfate, and with a lower radiopacity due to the lower content of bismuth oxide in its composition [21, 32]. MTA-P, which is claimed to have a finer particle size than currently available MTA brands, has been provided with either water or a hydrosoluble gel to improve its washout resistance (Avalon Biomed). A number of previous studies evaluated the fracture resistance of teeth completely filled with MTA, and conflicting findings were obtained. Fracture resistance of the teeth filled with MTA was found to be higher than those filled with CH [23, 33] or those filled with gutta-percha [2]. On the contrary, there have been some studies reporting that MTA did not have a significant effect on root strengthening [18, 24]. Tuna et al. tested the fracture resistance in human immature premolars using two different brands of MTA (MTA-A and MTA-PR), BA and CH [16]. Although MTA-A demonstrated significantly higher fracture resistance than MTA-PR, the difference did not reach statistical significance. In the present study, recent MTA brands MTA-P and MTA-A were tested, and MTA-P exhibited significantly higher fracture resistance than MTA-A. This result might be explained by the results of a recent investigation which has indicated that MTA-Plus (with either water or hydrosoluble gel) has a significantly lower washout compared to MTA-A [34].

In conclusion, the results of this in vitro study suggest that BA-filled immature teeth have higher fracture resistance than other groups at 24 months. In addition, the teeth filled with MTA-A demonstrated significantly lower strength to fracture in comparison with other groups. Considering the risk of cervical root fracture in the long term, it seems that BA could be a promising material. Further research is necessary to validate the findings obtained in the present study.

## Figures and Tables

**Table 1:** Manufacturer names and composition of the root canal filling materials used in the study.

Material	Company	Major chemical compounds
BioAggregate	DiaDent Group International, Canada	Tricalcium silicate, dicalcium silicate, tantalum pentoxide, calcium phosphate monobasic, amorphous silicon oxide

MTA-Angelus	Angelus, Londrina, PR, Brazil	Tricalcium silicate, dicalcium silicate, tricalcium aluminate, tetracalcium aluminoferrite, bismuth oxide

MTA-Plus	Avalon Biomed Inc. by Prevest Denpro Limited, India	Tricalcium silicate, dicalcium silicate, bismuth oxide, calcium sulfate, silica

EndoSequence Root Repair Material	Brasseler, USA	Tricalcium silicate, dicalcium silicate, zirconium oxide, tantalum oxide, calcium phosphate monobasic, calcium hydroxide, filler and thickening agents

**Table 2:** Mean fracture strengths (MPa) of teeth treated with BA, MTA-A, MTA-P, and ERRM at 24-month period and intergroup comparison of difference in fracture strength.

Groups	Fracture strength		^+^ *p*
	Mean ± SD	Median	
^1^BA	20.46 ± 2.53	21.04 (15.78–23.31)	***0.005*** ^*∗∗*^
^2^MTA-P	18.88 ± 5.13	17.45 (11.25–25.37)	
^3^MTA-A	14.12 ± 1.99	13.61 (11.71–18.02)	
^4^ERRM	17.65 ± 4.28	16.66 (12.57–26.37)	

1-2 ^†^ *p*	***0.739***		
1-3 ^†^ *p*	***0.001*** ^*∗∗*^		
1-4 ^†^ *p*	***0.063***		
2-3 ^†^ *p*	***0.029*** ^*∗*^		
2-4 ^†^ *p*	***0.579***		
3-4 ^†^ *p*	***0.035*** ^*∗*^		

^+^Kruskal-Wallis test; ^†^Mann-Whitney *U* test; ^*∗∗*^ *p* < 0.01; ^*∗*^ *p* < 0.05.
